# Equity-Oriented Design Processes and Evaluation of Digital Health Technologies for Black Communities Beyond Usability: Scoping Review

**DOI:** 10.2196/88995

**Published:** 2026-07-20

**Authors:** Myesha A Senior, Kejah Bascon, Myrtede Alfred, Enid Montague

**Affiliations:** 1 Department of Mechanical and Industrial Engineering, Faculty of Applied Science and Engineering, University of Toronto, 5 King’s College Road, Toronto, ON, M5S 3G8, Canada, 1 416-978-3040

**Keywords:** design, digital health equity, evaluation, digital health, Black-centered design, digital determinants of health, human-centered design

## Abstract

**Background:**

Black communities face disproportionate burdens of health disparities such as chronic disease, maternal morbidity, and barriers to accessing quality care. Digital health technologies (DHTs) are increasingly promoted as tools to reduce health disparities through access to care. However, the extent to which equity-oriented design approaches have been applied to address the needs of Black communities remains unclear.

**Objective:**

This scoping review aims to examine (1) current literature on DHTs designed to address health equity concerns among Black communities, (2) design approaches and evaluation mechanisms leveraged, and (3) the potential of the digital determinants of health (DDoH) framework to inform an equity evaluation of these tools.

**Methods:**

Guided by the PRISMA-ScR (Preferred Reporting Items for Systematic Reviews and Meta-Analyses Extension for Scoping Reviews), in April 2026, 3 online databases were searched (PubMed, Scopus, and Web of Science). Eligible studies were required to be published in English and report on the use of design approaches to develop DHTs for Black health equity concerns. Keywords related to digital health, equity, and design approaches were searched. No restrictions were placed on the publication date. Gray literature was searched using Google Scholar, and a reverse search was conducted through citation scanning. Identified studies were managed in Covidence with 2 independent reviewers. Data from included studies were extracted and mapped using tabular, graphical, and narrative synthesis.

**Results:**

Thirty-four studies were included in this review. Findings revealed a significant increase in publications over the past 5 years, with most studies conducted in the United States (25/34, 73.53%). HIV prevention or management was the most common health equity focus (9/34, 25.46%) with mobile apps as the primary technology (15/34, 44.12%). Most studies implemented a user-centered design (11/34, 32.35%) or a human-centered design (5/34, 14.71%) approach, while other studies used a combined or community-oriented approach. Two studies used an Afrofuturism approach to address broader dimensions of health equity, such as systemic racism and safety. Few studies explicitly evaluated digital health equity; assessments were typically limited to usability or feasibility (13/34, 38.24%). The application of the DDoH framework highlighted limited acknowledgment of determinants such as digital literacy, shared device privacy, and algorithmic bias.

**Conclusions:**

Despite the rise in the use of explicit design approaches to develop DHTs for Black communities, the application and evaluation of these approaches remain inconsistent. This review calls for more deliberate integration of equity metrics within the design process to ensure equitable design from start to finish. Leveraging frameworks such as the DDoH to conduct an equity analysis holds promise in assisting designers with keeping equity at the forefront of the design process. Through this work, 5 recommendations were proposed to standardize the design lifecycle to contribute to meaningful DHT design for the Black community.

## Introduction

### Background

Despite decades of public health interventions, persistent racial inequities remain entrenched in health outcomes. Black communities, in particular, face disproportionate burdens of chronic disease, maternal morbidity, mental health challenges, and barriers to quality care [1-3]. Furthermore, Black communities continue to face fragmented care pathways, limited access to services, and inadequate coordination across the health care continuum [4,5]. In the digital era, mobile apps, web-based platforms, and telehealth have been championed as innovative solutions to expand access, enhance care coordination, and address these disparities [6,7]. However, these technologies have produced limited measurable improvements in Black health outcomes. Studies show lower adoption and sustained use of digital health technology (DHT) among Black populations, often linked to mistrust, cultural misalignment, and persistent systemic inequities [8-10]. These patterns raise a critical question: are current design and development processes equipped to engage meaningfully with the lived realities of Black communities? Typically, design approaches prioritize efficiency within the process but in doing so, designers and researchers overlook deeper equity considerations, resulting in digital tools that are technically functional but culturally disconnected and fail to address the structural drivers of health inequities [11].

In the late 20th century, human-centered design (HCD) emerged as a formalized approach to creating products, services, and systems that prioritize the needs, experiences, and contexts of the people they serve [12,13,14,15]. HCD has been applied in various fields from health care to transportation [16,17]. Its traditional applications often lack explicit attention to cultural context, structural inequities, and justice-oriented outcomes that are integral to fields such as health care [18]. While some variations of the framework emphasize empathy, it has historically generalized notions of “the user” that may simplify the lived realities of marginalized communities [18,19]. Emerging critiques highlight that without the intentional integration of equity, power redistribution, and community ownership, dismantling inherent inequities is almost impossible [20,21].

In parallel with the expansion of HCD, in recent years the field has seen a growing “race” toward techno-solutionism, the belief that complex social and structural issues can be effectively solved through technological innovation alone [11,21,22]. Designs developed with traditional design approaches, including HCD, have resulted in technologies and systems that create and exacerbate disparities, a phenomenon known as technology-induced health disparities [23]. For example, electroencephalograms, which are used to measure electrical activity in the brain, are less effective at detecting brain signals in Black patients because of differences in hair texture and hairstyles [24]. Also, pulse oximetry devices have been found to be less reliable for patients with darker skin tones [25]. While such tools may be readily available, they fall short when deeper systemic inequities and histories of exclusion remain unaddressed. In response, emerging scholars and practitioners are beginning to reframe the design of technologies through frameworks such as Black-centered design (BCD) and Afrofuturism [26-28].

BCD positions the Black lived experience, cultural knowledge, and community-defined priorities at the center of the design process, challenging deficit-based narratives and instead building from histories of resilience and innovation [28]. The core principles of BCD include (1) engaging design principles that explicitly foreground Black lives and bodies, (2) seeking opportunities to involve the Black community in technological design, and (3) embracing interdisciplinarity in design practices [27]. BCD represents a way of thinking that integrates Black culture, historical context, and lived experiences within the current paradigm to dismantle systemic inequalities. In many cases, BCD is applied via the design lens of Afrofuturism, which envisions empowered futures for Black communities by centering justice and reimagining possibilities within the design process [28]. Scholars such as Harrington, Dillahunt, and Winchester III have begun to investigate the application of BCD and Afrofuturism to improve equity outcomes [27,29,30]. In the domain of health, Winchester III proposed the use of Afrofuturism to design a wearable health device to promote physical activity for Black women [27]. This case study resulted in a sketch that visualizes the centering of Black women in the design process. Winchester III suggested that Afrofuturism can offer a pathway beyond surface-level inclusion and toward transformative design practices, leading to technology that is not only functional but also fosters dignity, agency, and systemic change within its design [27].

Despite their transformative potential, BCDs remain underused in digital health design. Furthermore, at this time, there is limited evidence of a concrete understanding of the process of BCD within the health domain. This emphasizes the need to synthesize evidence on how both established and emerging design methods have been applied to develop digital health solutions for Black communities. Understanding this landscape is critical for identifying best practices, highlighting existing gaps, and informing future equity-centered innovation and evaluation. To assess equity consideration within the design process of DHTs, it is imperative that we explore the explicit implementation of digital health equity frameworks such as the digital determinants of health (DDoH) framework. According to Richardson et al [31], this framework can be used to assess digital health equity considerations and determinants across 4 levels: individual, interpersonal, community, and societal. This review aims to build on these critical evolutions in design practice, as we recognize that advancing health equity, particularly in Black communities, requires approaches that move beyond surface-level user engagement.

### Objectives

The objectives of this scoping review aim to examine (1) current literature on DHTs designed to address health equity concerns among Black communities, (2) design approaches and evaluation mechanisms leveraged, and (3) the potential of the DDoH framework to inform an equity evaluation of these tools. By examining the varying design approaches used with Black communities, this work seeks to gain a deeper understanding of the gap between traditional and equity-driven design approaches through the following research questions.

### Research Questions

To thoroughly address these objectives, we aimed to answer the following research questions:

- RQ1: What is the current state of research related to the design of DHT and tools aimed at addressing health equity concerns among Black communities?
- RQ2: How are the design approaches used in evaluating design output created for Black communities?
- RQ3: How can the DDoH framework be used to explicitly evaluate digital health equity outcomes?

## Methods

### Study Design

This scoping review was conducted and reported in accordance with the PRISMA-ScR (Preferred Reporting Items for Systematic Reviews and Meta-Analyses Extension for Scoping Reviews) guidelines (Checklist 1) [32]. To strengthen the transparency and reporting of our search process, we report our search in accordance with the PRISMA-S (Preferred Reporting Items for Systematic Reviews and Meta-Analyses Literature Search Extension Guidelines) [33].

### Study Protocol

Prior to conducting this review, an internal protocol was developed to outline and guide the review process. The research team developed a protocol based on the Joanna Briggs Institute (JBI) Manual for Evidence Synthesis template [34]. Although a protocol was developed to enhance methodological rigor and guide our work, it was not registered or published. Given the exploratory and iterative nature of the scoping review methodology, the protocol was intentionally treated as a flexible document that evolved alongside team discussions.

### Eligibility Criteria

Eligibility criteria for included studies focused on the design and development of digital health tools targeting Black communities. For the purposes of this review, the term Black community included Black communities across the African diaspora, including but not limited to African, Afro-Caribbean, and African American groups. Included studies were required to have a focus on addressing a specific health equity concern, whether explicit or implicit [35]. Furthermore, studies need to focus on the design or development of a DHT intervention, which may include but is not limited to mobile apps, chatbots, wearables, websites, or other digital health–related technologies. Records that clearly did not meet eligibility criteria (eg, unrelated populations; interventions outside the scope of digital health; or purely commentary pieces) were excluded. Table 1 outlines our eligibility criteria based on the population, concept, and context framework from the JBI Manual for Evidence Synthesis [34].

**Table 1.** Population, concept, and context eligibility criteria.

PCC^a^	Inclusion	Exclusion
Population	Studies involving Black communities across the African diaspora, including but not limited to African, Afro-Caribbean, and African American populations.	Studies focusing on non-Black populations or where Black communities were not a primary population of interest.
Concept	Studies implementing a design approach to develop digital health technologies to address health equity concerns. Digital health technologies could include mobile apps, chatbots, wearable devices, web-based platforms, SMS interventions, or other digital health tools. Studies addressing health equity concerns affecting Black communities, whether explicitly stated or implicitly addressed within the design of the digital health intervention.	Studies not involving digital health technologies, or studies not focused on design, development, or evaluation of digital health interventions. Studies that were unrelated to health equity or digital health interventions.
Context	As this review focuses on the broad topic of equity, studies can take place in any geographic location and within any cultural context. No limitations on the geographical, social, or cultural context.	Not applicable.

a PCC: population, concept, and context.

### Information Sources

A preliminary search of the Cochrane Database of Systematic Reviews and the JBI Evidence Synthesis was conducted and at the time no systematic reviews or scoping reviews were underway as it relates to this topic. Database searches were initially searched on November 2024, then refined and expanded, with the final search being conducted in April 2026. Databases that were individually searched include PubMed, Scopus, and Web of Science. We also conducted a gray literature search on Google Scholar, conference proceedings, and a secondary search of cited references for included studies. Online or print sources were not purposefully searched for this review, nor were data sought by contacting authors, experts, or manufacturers. No time limits were placed on obtaining relevant publications that related to the review, so email alerts were initiated across databases and periodically reviewed potential matches prior to data charting.

### Search

Our search strategy was developed through an initial consultation with subject matter experts to identify relevant literature and keywords at the intersection of digital health, design approaches, and health equity. Keywords were iteratively developed by the research team and were modified and adapted based on other searches from methods identified from the scoping review of digital health design approaches by Evans et al [35] and the seminal paper on Afrofuturism by Winchester III [27], where he outlines equity-centered design approaches. Table 2 provides an overview of key search concepts and terms used in this review. These terms were combined via Boolean operators and adapted for database-specific syntax. Due to the wide variation in the conceptualization of Black, African, or Afro-Caribbean identity, the research team did not include search terms around the concept of Black. Instead, this term was manually screened during the search process based on the eligibility criteria. No restrictions or filters were placed on publication date, article type, or study location. A full search string for the databases can be found in Multimedia Appendix 1.

**Table 2.** Search concepts and terms.

Concept	Example terms	Boolean operator
Health equity	health equity; digital health equity; digital divide	OR
Digital health	mHealth; mobile app; chatbots	OR
Design approaches	human-centered design; design thinking; Black-centered design; Afrofuturism; liberatory design	OR
Final search combination	Concept 1 AND Concept 2 AND Concept 3	AND

### Selection of Sources of Evidence

All records retrieved from the database searches were imported and managed in Covidence (Veritas Health Innovation Ltd), a reference management software, where duplicates were removed prior to screening [36]. The screening process followed a 2-stage approach in line with scoping review best practices. In the first stage, 2 reviewers independently screened the titles and abstracts of all retrieved citations against the predefined inclusion and exclusion criteria. In the second stage, the full texts of potentially relevant articles were retrieved and assessed for eligibility by the same 2 reviewers. Discrepancies in screening decisions were resolved through discussion and consensus, with a third reviewer available for adjudication if needed. Throughout the process, the reasons for exclusion at the full-text stage were documented to ensure transparency. The screening workflow and the number of studies included at each stage are summarized in a PRISMA-ScR flow diagram, which provides a visual overview of the identification, screening, eligibility, and final inclusion stages of the review [32].

### Data Charting Process

Following study selection, the data were systematically extracted into an Excel spreadsheet developed by the research team. Data charting items were iteratively developed through discussion among the research team. Once consensus was reached on the final template, extraction was conducted iteratively, with 2 reviewers independently coding a subset of studies to refine the framework and resolve discrepancies with the assistance of a third research team member (MA or EM) as needed.

### Data Items

The final data extraction items included (1) citation information (author, year of publication, title, and country of origin); (2) study characteristics (study objectives and sample size); (3) health domain; (4) methods (design approach, population characteristics, and evaluation approaches); and (5) outcomes (key findings, gaps, and recommendations). To further assess how digital health interventions were developed and evaluated for Black communities, we implemented the DDoH framework as a digital health equity evaluation mechanism. Included studies were assessed and coded for the presence of relevant determinants across each level, such as digital literacy, self-efficacy, patient-tech-clinician relationships, implicit bias, community partnerships, design standards, and data considerations. Where possible, verbatim language from the studies was used to justify meeting criteria to ensure fidelity to the authors’ descriptions.

A formal critical appraisal of individual sources was not conducted for our study. The purpose of this review was to map the breadth, characteristics, and conceptual design approaches within the current literature rather than to assess quality or risk of bias. Given the heterogeneity of study designs and evidence types within the design field, excluding studies based on quality criteria would have limited the comprehensiveness of the evidence map. Accordingly, findings should be interpreted as descriptive of the landscape of research rather than evaluating intervention.

### Synthesis of Results

The final extracted data were then synthesized using tables, graphics, and a narrative synthesis approach. This approach allowed us to generate a descriptive map of design and evaluation practices based on our research questions. Thematic insights were drawn and mapped to highlight patterns, underexplored domains, and gaps in equity evaluation based on study design and DDoH analysis.

## Results

### Overview

**Figure 1.**
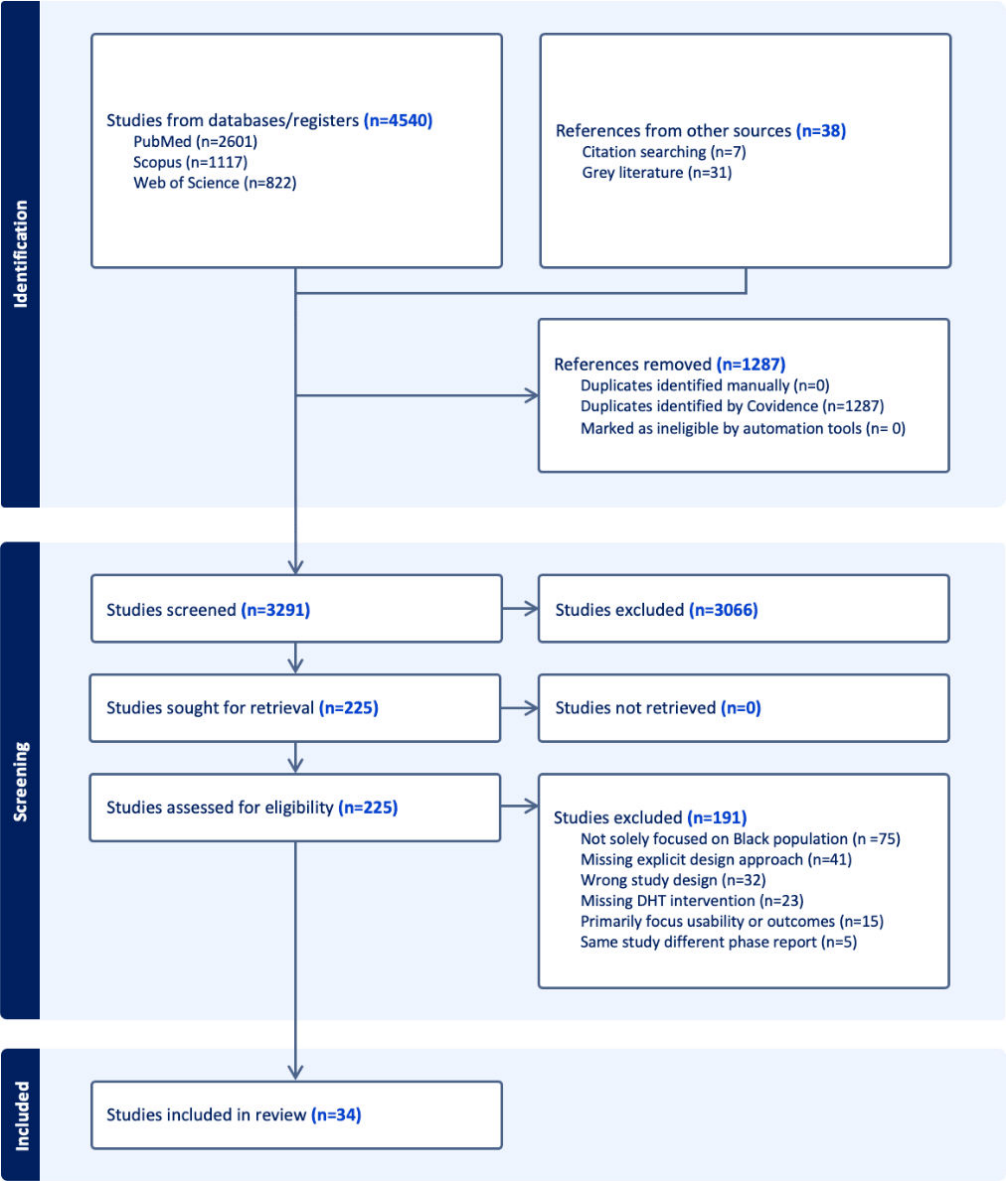
PRISMA (Preferred Reporting Items for Systematic Reviews and Meta-Analyses) 2020 flow diagram for new systematic reviews which included searches of databases, registers, and other sources.

A total of 34 studies were included in this scoping review after full-text screening [37-70]. The final number of studies at each stage of review is reported within the PRISMA (Preferred Reporting Items for Systematic Reviews and Meta-Analyses) flow diagram (Figure 1) [32]. These studies examined the use of various design approaches in the design and development of DHTs intended to address health outcomes for Black communities. The included studies were published between 2013 and 2026, with a notable increase in publications over the past 5 years, as shown in Table 3. Studies included a wide range of sample sizes across studies from 10 to 2019 participants, with most studies leveraging a qualitative (19/34, 55.88%) [38,41-45,47,51,53-55,57,58,62-64,66,67,70] or mixed methods research approach (15/34, 44.12%) [37,39,40,46,48-50,52,56,59-61,65,68,69].

**Table 3.** Study characteristics.

Author	Country	Methods	Design approach	Target population	DHT^a^ type	Health focus	Focus of evaluation
Amore et al (2023) [56]	United States	Mixed methods	User-centered design	Community: Black birthing parents (n=3), doulas (n=6), community organization representatives (n=4), and health care providers (n=6)	Web-based mHealth^b^ program	Maternal health related: maternal mortality risk assessment and educational tool	Quantitative: heuristic evaluation and beta testing. Qualitative: feedback session
Arueyingho et al (2025) [57]	Nigeria	Qualitative	Cross-cultural design	Community: Nigerian individuals living with type 2 diabetes, caregivers, and pharmacists (n=97)	Mobile app	Diabetes related: type 2 diabetes	Qualitative: feedback sessions which included think-aloud sessions and interviews to generate insights into the cross-cultural relevance and usefulness of the designed mobile app prototype
Baseman et al (2025) [58]	United States	Qualitative	Participatory speculative design	Black older adults (n=15), White (n=3)	Multimodal: mobile app, wearable, smart home	Diabetes related: diabetes broadly	Limited to no explicit evaluation: participants compare the 3 technologies based on which would be the best at the following: increasing diabetes knowledge, increasing foot health awareness, assessing foot health over time, helping with patient-doctor communication, and helping them discuss diabetes or foot care with trusted others.
Ben-Zeev et al (2021) [59]	Ghana	Mixed methods	User-centered design	Ghanaian community healers (n=42)	Mobile app	Well-being related: mental health	Quantitative: usability testing (feasibility, acceptability, and user burden). Qualitative: feedback interviews (open-ended questions about their experience navigating the tool, design preferences, and assessing content)
Blazey et al (2023) [37]	United States	Mixed methods	Human-centered design	African American women (n=10)	Mobile app	Cancer related: breast cancer	Quantitative: usability, feasibility, acceptance, and preference testing. Qualitative: formative feedback
Bosley et al (2022) [38]	United States	Qualitative	Afrofuturism; Healing Justice	African American community (n=10)	Multimodal: mobile app, physical resources	Well-being related: community trauma	Qualitative methods: focus groups, interviews, and observations related to understanding how participants felt about adapted videos
Brewer et al (2023) [39]	United States	Mixed methods	Culturally tailoring; CBPR^c^	African American community (n=45)	Multimodal: mobile app, wearable technology (wireless BP monitor)	Cardiovascular related: hypertension	Quantitative: ranking analysis based on 4 criteria: feasible, viable, desirable, and speed outcome measurement
Bruns (2021) [40]	South Africa	Mixed methods	Human-centered design	Black African men (n=2019)	Mobile app	HIV related: HIV intervention	Quantitative: acceptability, feasibility, usability, and usefulness assessed (some race or culture-based questions). Qualitative: formative feedback on preference
Chandler et al (2020) [41]	United States	Qualitative	Community-engaged	African American women (n=23)	Mobile app	HIV related: HIV prevention	Quantitative: usability testing (ease of use and acceptability). Qualitative: feedback from pilot test
Chandra et al (2024) [66]	United States	Qualitative	Co-design	African American community (n=11)	Mobile app	Cardiovascular related: cardiovascular broadly	Qualitative: initial feedback on design session and mood board outputs
Clement et al (2023) [67]	United States	Qualitative	User-centered design	African American men (n=24)	Mobile app	HIV related: HIV prevention	No explicit evaluation
Clifford et al (2022) [69]	United States	Mixed methods	Co-design; user-centered design; design thinking; community-based participatory design	African American young adults (n=505)	Multimodal: mobile app and wearables	Cardiovascular related: cardiovascular broadly	Quantitative: recorded number of downloads, number of users regularly uploading data (ie, at least 7 times a week), and types of data most frequently uploaded to app.
Evans et al (2016) [42]	United Kingdom	Qualitative	Social marketing design; CBPR	African community (n=48)	SMS text messages	HIV related: HIV prevention	No explicit evaluation
Huang (2024) [43]	United States	Qualitative	Participatory design with an integrated user-centered design and design thinking approach	Black older adults (n=28)	Virtual reality	Well-being related: Emotional well-being	No explicit evaluation
Isler et al (2019) [44]	Burkina Faso	Qualitative	Human-centered design	Black African pregnant and lactating mothers (n=36)	Video-based or web-based mHealth program	Maternal health related: maternal nutrition	Quantitative: usability testing. Qualitative: formative feedback on preference
Jefferson et al (2026) [70]	United States	Qualitative	Design thinking process augmented with the Public Health Critical Race Praxis	Community: lactation professionals (n=16), parents (n=7)	Mobile app	Maternal health related: breastfeeding	No explicit evaluation (future work)
Le et al (2018) [45]	United States	Qualitative	Community-engaged	African American women (n=15)	SMS text messaging	Cancer related: cervical cancer screening	Quantitative: usability testing. Qualitative: formative insights
Morse et al (2021) [46]	Tanzania	Mixed methods	Human-centered iterative design framework	Black African (n=31)	Multimodal: mobile app and web-based program	Cancer related: patients living with cancer	Qualitative: cognitive response testing for recommendations and initial usability and feasibility testing with end user
Musumbulwa et al (2026) [68]	Canada	Mixed methods	Community-based research; user-centered design	Black community members (n=6) and service provider (n=4)	Mobile health app	HIV related: HIV prevention	Quantitative: post study survey to evaluate usability, functionality, and motivational impact. Qualitative: reflecting on perspectives of app design, content, and persuasive potential
Newton Jr et al (2019) [65]	United States	Mixed methods	User-centered design	African American men (n=45)	Mobile app	Cardiovascular related: physical activity	Quantitative: usability and feasibility testing (SUS^d^ scale, user friendliness, satisfaction survey, and app usage)
Nias et al (2022) [47]	United States	Qualitative	Afrofuturism; design justice	African American community (n=not reported)	Wearable	Well-being related: physical and physiological protection for at-risk populations	No explicit evaluation
Olajide et al (2026) [64]	Australia	Qualitative	Co-design	African migrant women (n=7) and health care workers (n=5)	Multimodal: mobile app, web-based program	Maternal health related: pregnancy nutrition	No explicit evaluation
Patchen et al (2020) [48]	United States	Mixed methods	User-centered design; CBPR	African American parents or pregnant and postpartum women (n=25)	Mobile app	Maternal health related: breastfeeding	No explicit evaluation
Plant et al (2024) [49]	United States	Mixed methods	Human-centered design	Young Black gay and bisexual men (YBGBM; n=302) and providers (n=45)	Video-based and web-based mHealth program	HIV related: HIV intervention	No explicit evaluation
Popowski et al (2025) [63]	United States	Qualitative	User-centered design	African American adults (n=25)	SMS text messaging	Well-being related: mental health	No explicit evaluation
Resnick et al (2022) [50]	United States	Mixed methods	User-centered design, patient-led iterative process	African American patients (n=33)	Mobile app	Cancer related: cancer prevention- primary care	No explicit evaluation
Robles et al (2022) [51]	United States	Qualitative	Community-based participatory design	African American women (n=30)	Mobile app	Cardiovascular related: hypertension	Quantitative: assessing feasibility, patient satisfaction, and efficacy. Qualitative: formative feedback on design
Soehnchen et al (2023) [62]	Kenya	Qualitative	Intercultural user-centric design; double diamond; design thinking	Kenyan women (n=12) and experts (n=5)	Web-based app	Maternal health related: maternal health broadly	Qualitative: requirements engineering analysis
Tesema et al (2023) [52]	United States	Mixed methods	User-centered design	African American community (n=34)	Mobile app	HIV related: HIV prevention	No explicit evaluation
Thomson et al (2024) [53]	United States	Qualitative	Behavioral design thinking approach	Black men (n=105)	Mobile app	Cancer related: colorectal cancer screening	Qualitative: feedback session with distinct motivation scenarios
Veinot et al (2013) [54]	United States	Qualitative	User-centered design	African American community (n=75)	Multimodal: mobile app and web-based mHealth program	HIV related: HIV prevention	Qualitative: formative feedback (perceived benefits and concerns, message content, and intervention structure)
Vilaro et al (2021) [61]	United States	Mixed methods	User-centered design	Black women (n=53)	Web-based app	Cancer related: colorectal cancer	Quantitative: questionnaire of perceptions of virtual health agent and application. Qualitative: perceptions of source cues and navigability
Williamson et al (2021) [60]	United States	Mixed methods	Community-based participatory research; trust-centered design	Community: n=315; 280 African American participants	Multimodal: web-based program and social media accounts	HIV related: HIV outreach, prevention, education	Quantitative: self-reported uptake, engagement (surveys), usage (server logs). Qualitative: interviews for factors affecting usage
Zhou et al (2024) [55]	United States	Qualitative	Culturally tailored	African American community (n=34)	Video-based and web-based mHealth program	Well-being related: insomnia	Quantitative: usability testing and heuristic analysis (functionality, usability, and emotional resonance with this demographic)

a DHT: digital health technology.

b mHealth: mobile health.

c CBPR: community-based participatory research.

d SUS: System Usability Scale.

### Current State of Black Health Design Research (RQ1)

#### Study Characteristics

**Figure 2.**
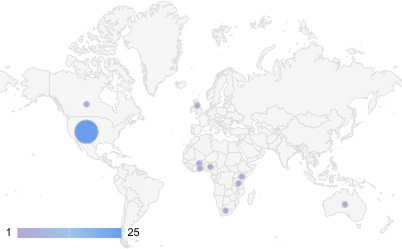
A geographical map illustrating the geographic distribution of included studies.

All included studies focused on Black, African American, or African populations. Most studies (20/34, 58.82%) [38,39,42,43,46,47,50,52,54-60,63,66,68-70] focused on addressing the health needs of the Black or African community as a whole, whereas 9 studies focused specifically on the health needs of women [37,41,44,45,48,51,61,62,64], with only 5 studies explicitly targeting male participants [40,49,52,65,67]. Most studies were conducted in the United States (25/34, 73.53%) [37-39,41,43,45,47-56,58,60,61,63,65-67,69,70], with the remaining studies conducted outside of the United States, with the majority conducted in the continent of Africa (n=6; Figure 2) [40,44,46,57,59,62].

#### DHT Characteristics

**Figure 3.**
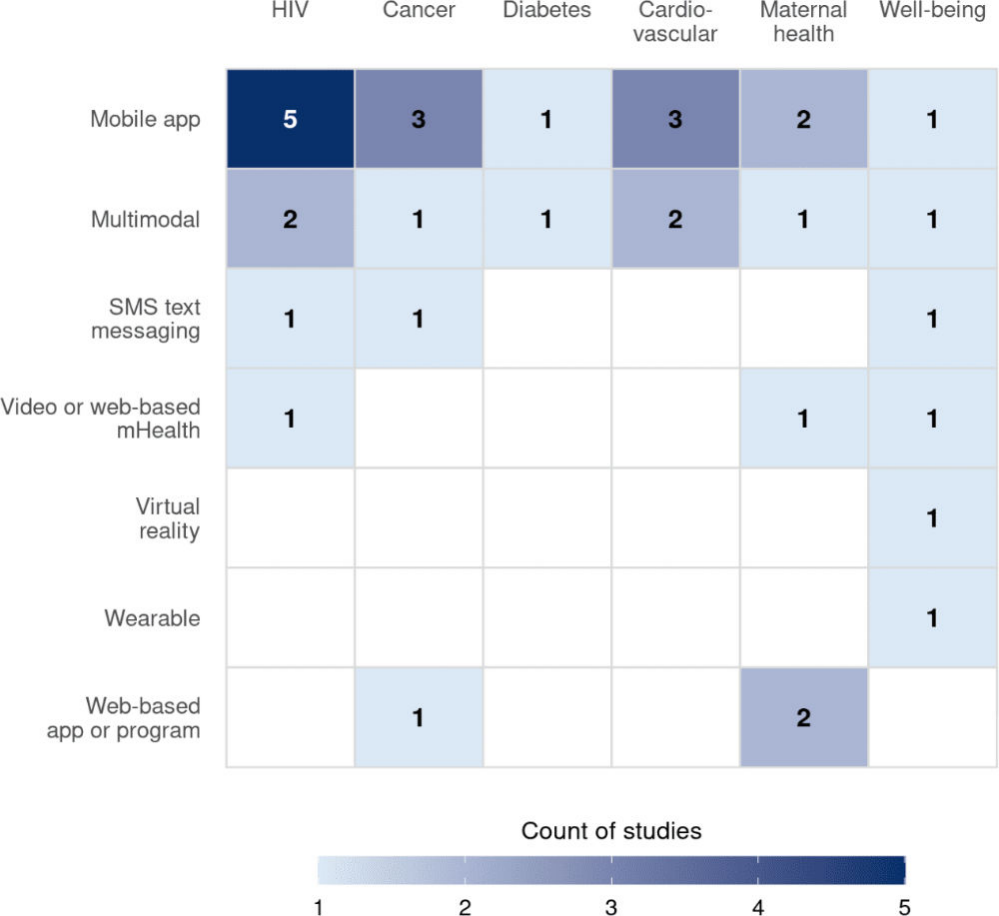
Heat map showing the distribution of digital health technology types across health domains in the included studies (darker blue cells indicate a higher number of studies addressing a given health domain using a particular digital technology modality).

The most common health equity domain addressed was HIV prevention or management (9/34, 25.46%) [40-42,49,52,54,60,67,68], followed by cancer [37,45,46,50,53,61], maternal health [44,48,56,62,64,70], and well-being–related health topics [38,43,47,55,59,66] (6/34, 17.64%, respectively). Of studies focusing on well-being, some explored more complex health and well-being concerns, such as the mental or emotional impacts of police brutality [38,47] and nostalgia used to improve emotional well-being in Black older adults [43]. Mobile apps were the most frequently used digital health modality across studies (15/34, 44.12%) [37,40,41,47,50,51,53,57,59,66-68,70], particularly for HIV-related (5/34, 14.71%) [40,41,52,67,68] and chronic illness (7/34, 20.59%) [37,50,51,53,57,65,66] (Figure 3). Multimodal technology (a combination of DHTs) was also used across several conditions, whereas emerging technologies such as virtual reality and wearable devices were primarily used in studies focused on well-being.

### Implementation and Evaluation of Design Approaches (RQ2): Implemented Design Approaches

User-centered design (UCD; 11/34, 32.35%) [48,50,53,54,56,59,61,63,65,67,68] was the most used design approach, followed by HCD (5/34, 14.71%) [37,40,44,46,49]. Other studies leveraged community-oriented approaches such as community-based participatory design [52] and community-engaged approaches [41,45]. Only 3 included studies used speculative design approaches [38,47,58][31,40,51] with 2 studies leveraging Afrofuturism as an explicit BCD design approach [38,47]. Table 4 maps the application of the design approaches and evaluation methods for included studies.

**Table 4.** Grouped design approaches and evaluation.

Design approach and study	Overview of the design process	Focus of evaluation
Human-centered design (n=5)		
Blazey et al (2023) [37]	Phase 1: intervention design and development Phase 2: user testing Phase 3: pilot study	Quantitative: usability, feasibility, acceptance, and preference testing Qualitative: formative feedback
Isler et al (2019) [44]	Phase 1: FGD^a^ - (to understand the perception of original videos and gain insights to adapt them) Phase 2: FGD, in-depth interviews, and observations	Qualitative methods: focus groups, interviews, and observations related to understanding how participants felt about adapted videos
Bruns (2021) [40]	Evidence Empathy Eureka Execution Evolution	Quantitative: ranking analysis based on 4 criteria: feasible, viable, desirable, and speed outcome measurement
Plant et al (2024) [49]	Stage 1: ideation Stage 2: rapid prototyping Stage 3: user feedback	Quantitative: acceptability, feasibility, usability, and usefulness assessed (some race- and culture-based questions) Qualitative: formative feedback on preference
Morse et al (2021) [46]	Stage 1: establishing the study team Stage 2: defining a set of app design requirements Stage 3: defining user requirements Stage 4: creating the app prototype Stage 5: expert UX^b^ review Stage 6: testing the prototype	Quantitative: usability testing (ease of use and acceptability) Qualitative: feedback session
User-centered design (n=11)		
Veinot et al (2013) [54]	Limited explicit overview of the design process FGDs	No explicit evaluation
Thomson et al (2024) [53]	Limited explicit overview of the design process Surveys and FGDs	No explicit evaluation
Patchen et al (2020) [48]	Phase 1: identify ideal technological components and content of an mHealth^c^ intervention Phase 2: determine the usability of a prototype based on phase 1 findings	Quantitative: usability testing Qualitative: formative feedback on preference
Resnick et al (2022) [50]	Stage 1: solicit feedback from participants on paper mock-ups of the app Stage 2: the study team walked participants through a beta version of the app on a smartphone	Quantitative: usability testing Qualitative: formative insights
Amore et al (2023) [56]	Phase 1: content development (desk research) Phase 2: feedback session (focus groups) and heuristic evaluation. Phase 3: algorithm design, tool design and development, then beta testing with experts	Quantitative: heuristic evaluation and beta testing Qualitative: feedback session
Ben-Zeev et al (2021) [59]	On-site interviews and observations at prayer camps in Ghana Collaborative co-design session with healers to gather rapid feedback regarding possible design approaches Content development and prototype system build-out Usability assessment with target end users.	Quantitative: usability testing (feasibility, acceptability, and user burden) Qualitative: feedback session
Vilaro et al (2021) [61]	Limited explicit overview of the design process FGDs reviewing printed prototypes (2) or interactive prototypes (6) Think-aloud interviews (6)	Quantitative: questionnaire of perceptions of virtual health agent and application. Qualitative: perceptions of source cues and navigability
Popowski et al (2025) [63]	Limited explicit overview of design process FGDs and interviews to understand user needs	No explicit evaluation
Newton Jr et al (2019) [65]	Limited explicit overview of design process FGDs, usability testing, feasibility testing	Quantitative: usability and feasibility testing (SUS^d^, user friendliness, satisfaction survey, and app usage)
Clement et al (2023) [67]	Limited explicit overview of design process FGDs	No explicit evaluation
Musumbulw a et al (2026) [68]^e^^,^^f^	Stage 1 (previous study): collected feedback on the most relevant structural, institutional, and individual barriers hindering engagement in HIV prevention initiatives Stage 2: implemented persuasive strategies Stage 3: app development, Stage 4: formative evaluation	Quantitative: survey to evaluate usability, functionality, and motivational impact Qualitative: formative feedback on perspectives of app design, content, and persuasive potential
Community-engaged (n=2)		
Le et al (2018) [45]	Community engaged process: Conducting formative research for intervention development Setting the study in the community, at an agreed-upon location and time of convenience to the study participants Securing buy-in and recruitment or retention support from pastors and community health advisors Forming a community advisory board; and Building in and carrying out member checks throughout the study	Qualitative: cognitive response testing for recommendations and initial usability and feasibility testing with end user
Chandler et al (2020) [41]	Limited explicit overview of the design process FGDs	No explicit evaluation
Afrofuturism (n=2)		
Boosley et al (2022) [38]	Limited explicit overview of the design process: Guided by Afrofuturist Feminism, Healing Justice, and Tenets of Joy and designing Civic Engagement workshops, surveys, and workbook activities were conducted.	No explicit evaluation
Nias et al (2022) [47]	Guided by design justice principles: Design to sustain, heal, and empower our communities Prioritize design’s impact on the community Work toward nonexploitative solutions that reconnect us to the earth and to each other Look for what is already working at the community level Work toward sustainable, community-led, and controlled outcomes	No explicit evaluation
Cultural tailoring (n=2)		
Zhou et al (2024) [55]	Used the Common Strategies for Enhancing Cultural Appropriateness to guide cultural tailoring [71]: Peripheral: designing materials to appeal to the patient group Evidential: enhancing relevance of the health issue by showing epidemiological evidence Linguistic: providing materials in the primary language of the target group Constituent-involving: drawing from the experiences of the patient population Sociocultural: using cultural values, beliefs, and behaviors to provide context and meaning to the health messages	No explicit evaluation
Brewer et al (2023) [39]	Phase 1: solicit input through focus groups to refine an existing culturally tailored mHealth app Phase 2: a single-arm pre-post intervention pilot study assessing feasibility and patient satisfaction	Quantitative: assessing feasibility, patient satisfaction, and efficacy. Qualitative: formative feedback on design
Design thinking (n=3)		
Olajide et al (2026) [64]	Empathize: qualitative interviews Define: synthesize needs and plan or prep workshop Ideate: web-based delivery of 2 workshops Prototype: rank top 4 resources Test: future work	No explicit evaluation (future work)
Thomson et al (2024)^g^ [53]	Empathize with users and their behavior change needs Define user and behavior change requirements Ideate user-centered features and behavior change content Prototype a user-centered solution that supports behavior change	No explicit evaluation
Jefferson et al (2026)^g^ [70]	Design thinking process: Empathy Define Ideate Prototype Test Critical Race Praxis focus areas: Contemporary patterns of race relations Knowledge production Conceptualization and measurement Action	No explicit evaluation (future work)
Co-design (n=1)		
Chandra et al (2024) [66]	Limited explicit overview of the design process: The design team led several rounds of iterative brainstorming to craft ideas about the storytelling and cultural aspects of the digital application design.	Qualitative: initial feedback on design session and mood board outputs
Robles et al (2022) [51]	Discovery interviews Empathy mapping Design sprint Storyboarding and feedback session Paper prototyping and feedback session	Qualitative: feedback session with distinct motivation scenarios
Social marketing design (n=1)		
Evans et al (2016) [42]^e^^,^^f^	Draft messages and team review (FGDs) Pretesting using Health Behavioral Model with members of the African community Research evidence to inform modifications Team agreement and review	Qualitative: formative feedback (perceived benefits and concerns, message content, and intervention structure)
Participatory design with an integrated user-centered design and design thinking approach (n=1)		
Huang (2024) [43]^f^	Empathize Define Ideate Prototype Test	Quantitative: usability testing and heuristic analysis (functionality, usability, and emotional resonance with this demographic)
Co-design; user-centered design; design thinking (n=1)		
Clifford et al [69]^e^,^f^	Limited explicit overview of the design process: CBPR and UCD^h^ to engage the end user in identifying environmental factors of CVD^i^ risk Health Belief Model to increase the understanding and ownership of the disparity that exists Design-thinking to explore solutions to problems associated with the sustained use of mHealth technologies Aligning identified requirements with the social cognitive theory of mass communication	Quantitative: recorded number of downloads, number of users regularly uploading data (ie, at least 7 times a week), and types of data most frequently uploaded to app.
Participatory speculative design (n=1)		
Baseman et al (2025) [58]	Limited explicit overview of the design process: 4 participatory speculative design workshops: group-based speculative design activity centering 3 modalities of ubiquitous diabetes technologies.	Qualitative: feedback (comparison of 3 technologies ability to (1) increase diabetes knowledge and (2) foot health awareness, (3) assess foot health over time, (4) help with patient-doctor communication, and (5) help with discussion of diabetes or foot care with trusted others.
Cross-cultural design (n=1)		
Arueyingho et al (2025) [57]	Stage 1: use of design cards to define the scope of T2D^j^ care Stage 2: culturally appropriate role-playing exercise Stage 3: use of probes to inspire culturally and contextually relevant conversations Stage 4: sketching, wireframing, and prototyping sessions Stage 5: feedback sessions	Qualitative: feedback sessions (think-aloud sessions and interviews to generate insights into the cross-cultural relevance and usefulness of the designed mobile app prototype)
Intercultural user-centric design; double diamond; design thinking (n=1)		
Soehnchen et al (2023)^f^ [62]	Phase 1: product development, identifies user requirements, who the user is, and what should be developed. Phase 2: defines the concept, creating a cross-cultural design philosophy to adapt to the respective culture. Phase 3: drafts and specifies the product as a detailed requirements engineering analysis by including the respective culture and end user. Phase 4: software development.	Qualitative: requirements engineering analysis
Trust-centered design (n=1)		
Williamson et al (2021) [60]^e^^,^^f^	Limited explicit overview of the design process: Interviews and surveys (centering trust in the development of intervention and leveraging trust-related design requirements)	Quantitative: self-reported uptake, engagement (surveys), and usage (server logs) Qualitative: interviews for factors affecting usage

a FGD: focus group discussion.

b UX: user experience.

c mHealth: mobile health.

d SUS: System Usability Scale.

e Implemented listed design method in combination with community-based participatory research approach.

f Combination of design approaches.

g Expanded version of design thinking approach.

h UCD: user-centered design.

i CVD: cardiovascular disease.

j T2D: type 2 diabetes.

Included studies approached the HCD process with varying levels of user engagement. Two studies engaged participants throughout the entire HCD process, including the cocreation of these prototypes [40,49], whereas others leveraged participant insights from the needs assessments and then developed prototypes based on their design requirements [44,46]. Studies that used a design thinking approach typically explicitly defined each phase (empathize, define, ideate, prototype, and test), engaged with participants throughout most phases but did not achieve the evaluation stage with participants within the study [50,64,70]. Of those that leveraged the design thinking approach, 2 expanded this approach using behavioral design and Critical Race Praxis to explicitly ensure actionable and culturally relevant outcomes [50,70].

Six studies were used in combination with others, with the majority of the studies combining methods with design thinking, UCD, and co-design [42,43,60,62,68,69], which limited the explicit explanation of the design process. Community-based participatory research (CBPR) approaches were often combined with methods, such as cultural tailoring, trust-centered design, UCD, and social marketing design [39,42,60,68,69]. These studies used CBPR methods to directly interact with participants during the needs assessment stage.

Two studies that used Afrofuturism varied in the description of the design process and used guiding principles (Healing Justice and Design Justice principles) during the co-design process [38,48]. One study engaged participants throughout the speculative design process with workshops [38]. The other study did not report direct participant engagement; rather focused on the creation of the prototype being grounded in current systemic issues faced by the community [47].

### Evaluation of Equity Outcomes

Although all studies aimed to implicitly address health disparities facing this community, only a small subset explicitly defined terminology such as “health equity” or “racial equity” in framing their work (n=4) [38,47,48,70]. Two studies that incorporated Afrofuturism discussed the value of centering Black experiences in the design process to achieve more just and inclusive outcomes [38,47]. As mentioned, Jefferson et al [70] merged design thinking with the Public Health Critical Race Praxis, which focuses on confronting the structural mechanisms precipitating and perpetuating health inequities throughout the design process, but evaluation from this perspective was described as future work. Furthermore, many studies did not describe specific evaluation methods used within the design process (11/34, 32.35%) [38,41,47,50,53-55,63,64,67,70]. Two of these studies mentioned evaluation as future work of the study [64,70]. Among the studies that were evaluated, the majority incorporated usability testing, feasibility, and acceptability surveys, or open-ended user feedback on functionality and other characteristics (13/34, 38.24%) [37,40-42,44-46,52,55,56,59,65,68]. Notably, only one study assessed changes in health outcomes [39], and one included longitudinal follow-up data beyond initial pilot testing [40].

### Deeper Equity Analysis With DDoH Framework as an Equity Evaluation (RQ3)

Across the studies in our review, several studies referenced determinants across the 4 DDOH levels: individual, interpersonal, community, and societal (Multimedia Appendix 2). The heaviest concentration of evaluation activities was clustered at the individual level (self-efficacy, attitudes toward technology, and access), with community norms and partnerships also frequently described. In contrast, societal determinants were discussed more sparingly (eg, algorithmic bias, data standards, and tech policy). Table 5 below maps the various papers against the DDoH factors.

**Table 5.** Summary of studies associated with DDoH^a^ factors.

Digital determinants	Studies
Individual level	
Attitudes toward use	[37-45,47-63,66-68,70]
Digital literacy	[39,41,43,44,46,48,53,57-59,64,68,69]
Digital self-efficacy	[37-39,41-45,47-54,56-58,68]
Technology access	[37,39-50,52-65,67,68]
Interpersonal level	
Implicit tech bias	[37,38,41-43,47-49,52-58,70]
Interdependence (eg, shared devices)	[39,41,46,52,54]
Patient-tech-clinician relationship	[40-42,44,46,49,52,54,56-58,61,63,64,67-69]
Community level	
Community infrastructure	[37-39,41-45,47-55,57-60,62,67,68]
Health care infrastructure	[40,41,44-46,49,62,63,68,69]
Community tech norms	[37-50,52,54,56-63,66-68]
Community partnerships	[39-46,48-50,53-60,62,66,68-70]
Societal level	
Tech policy	[40,41,47,49,50,52,54,59,69]
Data standards	[41,47,49,50,52,54,56-60,63,67-69]
Design standards	[38-41,46,47,49,50,52-54,56,57,59-62,65,68,69]
Social norms and ideologies	[37-70]
Algorithmic bias	[47,57]

a DDoH: digital determinants of health.

### Individual Level

All studies assessed individual readiness in some form. Attitudes toward use appeared across nearly all the studies (30/34, 88.23%) [37-45,47-63,66-68,70], as did digital self-efficacy (20/34, 58.82%) [37-39,41-45,47-54,56-58,68]. Other proxies, such as technology access (29/34, 85.29%) [37,39-50,52-65,67,68], were commonly noted through device or connectivity availability. An example of this can be seen in the work of Le et al [45], where they developed the CervixCheck project, a spiritually based SMS intervention for church-attending African American women. The results showed that the participants found SMS text-messaging systems to be the most accessible and acceptable form of technology for this community while boosting digital self-efficacy. Notably, digital literacy was explicitly measured or discussed in far fewer cases (13/34, 38.23%) [39,41,43,44,46,48,53,57-59,64,68,69].

### Interpersonal Level

Interpersonal dynamics was the second most prominent level of DDoH, as many studies mentioned one factor across this level. Most prominently, these factors included implicit technology bias (16/34, 47.06%) [37,38,41-43,47-49,52-58,70] and patient-technology-clinician relationship factors (17/34, 50%) [40-42,44,46,49,52,54,56-58,61,63,64,67-69]. For example, Morse et al [46] developed a mobile-Palliative Care Link web and mobile app for outpatient symptom assessment and coordination focused on ensuring usability and connectivity across patients, providers, and caregivers. A smaller subset addresses device interdependence (eg, shared phones or accounts, 5/34, 14.71%) [39,41,46,52,54]. One example of this consideration was a mobile app, developed by Blazey et al [37], which, for Black breast cancer survivors and their first-degree relatives, uses a human-centered approach to promote physical activity through dyads, culturally tailored content, and goal-setting features while also considering the use of shared devices in the household [37]. One study mentioned the limited access to personal devices due to geographical constraints within Kenya but never reengaged this topic when discussing design requirements [62].

### Community Level

Community-level determinants were often addressed through community technology norms (27/34, 79.41%) [37-50,52,54,56-63,66-68] and the presence of community partnerships (23/34, 67.65%) [39-46,48-50,53-60,62,66,68-70] or community infrastructure (21/34, 61.77%) [37-39,41-45,47-55,57-60,62,67,68] considerations during the development phase. For example, Patchen et al [48] developed a breastfeeding app for African American parents via community-based participatory methods, incorporating features such as peer discussion forums, local resources, and support person access to ensure cultural relevance and usability [48]. Similarly, Isler et al [44] adapted a suite of South African maternal nutrition videos for rural Burkina Faso through HCD, engaging both community health workers and mothers to refine content, language, and delivery to fit local realities. Few studies address health infrastructure within their design and development (10/34, 29.41%) [40,41,44-46,49,62,63,68,69].

### Societal Level

At the societal level, all studies engaged with social norms and ideologies through mention of cultural narratives, faith-based health promotion, peer support structures, and stigma surrounding sensitive conditions. Moderate attention was given to design standards (20/34, 58.82%) [38-41,46,47,49,50,52-54,56,57,59-62,65,68,69], while fewer studies focused on data standards (15/34, 44.12%) [41,47,49,50,52,54,56-60,63,67-69] and technology policies (9/34, 26.47%) [40,41,47,49,50,52,54,59,69]. Plant et al [49] highlighted how design features such as anonymity, membership controls, and rumor management are essential for fostering trust in an online HIV prevention intervention, illustrating how both interface design and data safeguards shape user confidence and engagement. Algorithmic bias appeared only twice (2/34, 5.88%) [47,57], for example, Nias et al [47] proposed an Afrofuturist wearable hoodie with embedded biometric sensors to provide protective algorithmic design and data sharing among Black communities during moments of distress. Conversely, Clifford et al [69] leveraged a UCD approach to develop an open-source privacy-oriented multimodal tool for cardiovascular health monitoring and intervention planning mentions but did not mention the consideration of algorithmic bias within the design process.

## Discussion

### Summary of Findings

This scoping review maps the current landscape of the design of DHTs for Black communities. A notable increase in publications over the past 5 years was observed, with the majority of studies focusing on designing mobile health apps. HIV was found to be the most prominent health equity concern within the included studies followed by cancer-related concerns. While the included studies reflect efforts to address racial health disparities through digital innovation, our review highlights the wide variation in the application of approaches to design and evaluate health equity within the digital domain. HCD and design thinking approaches often leverage step-by-step guidance to design with Black communities, but the level of user engagement and equity evaluation fluctuated between studies. Among the included studied BCD approaches, such as Afrofuturism, appeared less often in the literature, and design outputs were often not evaluated.

Among the included studies, few articulated a clear framework for evaluating health equity outcomes or digital health equity specifically. Although cultural relevance, trust-building, and stakeholder preferences are commonly discussed, the lack of formal evaluation methods, longitudinal data, and health equity outcome measures based on these criteria limits our understanding of the equity impacts and sustainability of these interventions. Usability, feasibility, and acceptability were the most common evaluation criteria, yet these approaches rarely make equity an explicit focus. An equity analysis conducted through the use of the DDoH framework revealed blind spots within the design of DHTs. Determinants such as digital literacy, interdependence (shared device), and algorithmic bias are not explicitly considered throughout the design and evaluation process, with less than half of studies acknowledging these elements.

### Comparison With Prior Work

While prior reviews have examined the use of HCD approaches in advancing health equity, none have critically assessed their impacts on health equity for Black communities or explored the application of Black-centered design approaches in their reviews [35]. Furthermore, to our knowledge, only one study to date has applied the DDoH framework as an analytical lens to evaluate the equity implications of design approaches in digital health [72].

Notably, this review demonstrates that research on DHTs designed for Black communities remains geographically concentrated and limited in scope. As mentioned, most included studies were conducted in the United States and focused primarily on HIV prevention or management. This distribution aligns with broader public health scholarship highlighting that digital health interventions targeting Black populations have historically emerged in response to diseases with well-documented racial disparities, particularly HIV or AIDS [73,74]. Prior reviews of mobile health interventions similarly report a concentration of research in infectious disease prevention and management [75,76]. While these efforts represent important contributions to addressing health inequities, this limited scope may inadvertently overshadow other highly prevalent conditions affecting Black populations, including cardiovascular disease, mental health, and diabetes [77]. Consequently, DHTs targeting these conditions may continue to rely on generalized one-size-fits-all HCD approaches that insufficiently account for the historical, cultural, and structural factors shaping health experiences in Black communities [18]. Suggesting that the potential of DHTs to address broader determinants of health in Black communities remains underexplored.

Our findings also reveal that human-centered and UCD approaches dominate the development of digital health tools for Black populations, while explicitly Black-centered frameworks remain rare. Human-centered, user-centered, and community-engaged approaches were commonly used to improve usability, cultural tailoring, and adoption of interventions, consistent with prior reviews emphasizing the importance of HCD and participatory design in health technology development [5]. CBPR approaches have long been recognized as essential for addressing health disparities by incorporating community expertise and fostering trust [78]. However, only a small number of studies explicitly incorporated frameworks such as Afrofuturism or BCD. This finding reflects broader critiques within design justice scholarship, which argue that many participatory approaches still operate within dominant design paradigms that do not fully address structural inequities or redistribute power within design processes [79]. Furthermore, speculative design approaches such as Afrofuturism have been critiqued for their speculative and conceptual orientation, which may limit their perceived applicability within traditional health research frameworks and potentially influence researchers’ willingness to engage with these approaches in digital health intervention design [80,81]. Recently, the study by Groeneveld et al [82] has proposed step-by-step guidance around implementing future-focused health care research, which may be expanded and used as guidance on how to engage and report speculative health research, such as Afrofuturism in a more systematic way. As such, participatory present-day approaches appear more widely adopted, but deeper epistemological shifts toward design approaches that center Black knowledge systems and Black health futuring are needed.

According to Egede et al [83], if we begin to prioritize key target areas linked to structural racism within the health care sector, these can be used to enhance health outcomes and achieve equity regardless of race, gender, or socioeconomic status. Uniquely, Afrofuturism design approaches within the review addressed more complex racial well-being concerns, such as police brutality and systemic oppression through provoking reflection on structural and systemic factors impacting the community as a design space similar to emergent works [84]. Furthermore, 2 studies leveraged cross-cultural design and intercultural UCD approaches, which may warrant further exploration within Black-centered design research [57,62]. This aligns with the emergent field of cultural ergonomics, defined by Smith-Jackson et al [85], which emphasizes the importance of designing systems that account for culturally mediated behaviors, values, expectations, and interactions across diverse contexts. These approaches acknowledge that Blackness or identity is shaped differently across geographic, cultural, and sociopolitical contexts, while emphasizing more intersectional and contextually grounded understandings of lived experience in the translation of community needs into design requirements [62]. Expanding on this work may strengthen efforts to develop culturally responsive DHTs that move beyond the representation of Black identity as a monolith and instead account for the diversity of experiences across the African diaspora.

Another notable finding from this review is the emphasis on usability, feasibility, and acceptability as primary evaluation outcomes for digital health interventions. Among the studies reviewed, most assessed factors such as user satisfaction, perceived usefulness, and engagement with technology, while very few evaluated downstream health outcomes, long-term behavioral change, or equity criteria. This pattern is consistent with broader digital health research, where early-stage pilot studies frequently prioritize feasibility and user experience over clinical or population-level impact [86]. Although such evaluations are important for ensuring that interventions are usable, limited outcome-based assessments and equity evaluation impact the ability to determine whether these technologies can ultimately bridge the disparity gaps for this community or further push the digital divide [87,88].

Critically, assessing the digital health equity of DHTs continues to be a pressing concern across the board for scholars [89]. Lyles et al [90] stated that to minimize the harms of digital tools, we must focus on proactive engagement, planning, and implementation to ensure the equitable deployment of DHTs. Emerging scholarship from cultural ergonomics calls for the explicit understanding of discrete events in which breakdowns occur due to mismatches between system design and culturally embedded expectations, communication norms, or values [91]. The findings of this review highlight the importance of considering digital health interventions through a multilevel lens to uncover these moments. By using the DDoH framework as a tool to further the equity analysis and considerations throughout the design process, we were able to identify blind spots and opportunities within the design of DHTs [31]. Across studies, determinants of digital health appeared across the individual, interpersonal, community, and societal levels, which highlights the rise in consideration of these factors. At the individual level, most studies assessed attitudes toward technology, self-efficacy, and access to digital devices, factors which have been widely identified as key predictors of technology use in digital health interventions [87,92]. However, far fewer studies have examined digital literacy or interdependence, suggesting a gap between measuring willingness to engage with technology and understanding the capabilities required to use these tools effectively [87]. This pattern suggests that while many evaluations ask whether people are able and motivated to use tools, fewer interrogate whether users possess the specific literacies required to navigate these tools when accessing them.

Interpersonal and community dynamics were also prominent across the literature. Many studies incorporated considerations such as patient-tech-provider relationships, peer support networks, and community partnerships in the design and implementation of interventions. These findings align with research demonstrating that trust, social relationships, and culturally embedded support systems are critical facilitators of digital health engagement in marginalized communities [93]. Faith-based institutions, community organizations, and peer networks have long served as trusted third spaces for health promotion in Black communities, and their integration into digital interventions reflects broader strategies for culturally responsive public health programming [45,78]. Few studies addressed health infrastructure within their design and development, highlighting a gap in consideration of this subdomain, as it focuses on community access to varying health care systems and tools.

Finally, at the societal level, nearly all studies engaged with broader social norms and cultural narratives shaping technology adoption. Issues such as stigma, privacy concerns, and the need for anonymity were particularly prominent in interventions targeting sensitive health conditions such as HIV. These findings are consistent with prior research showing that digital technologies can provide opportunities for discreet engagement with health services, particularly for stigmatized conditions [94]. However, the limited attention given to algorithmic bias, data governance, and structural inequities within digital infrastructures suggests that many studies have yet to fully engage with emerging concerns about the role of technology in reproducing systemic inequities [90,93]. Recent scholarship on digital health equity and algorithmic fairness emphasizes the inadvertent reinforcement of existing disparities if issues such as biased data, inequitable access, and culturally insensitive design practices are not addressed during development [95,96].

Taken together, these findings suggest that while digital health interventions for Black communities increasingly incorporate HCD or participatory design practices and multilevel considerations, important gaps remain in both conceptual framing and equity evaluation. The limited integration of explicitly Black-centered frameworks, the primary focus on usability evaluations, and the uneven attention to structural determinants of digital health highlight the need for more comprehensive and equity-driven approaches to technology design and implementation. Moreover, as Afrofuturist and Black-centered design paradigms continue to emerge, there is a significant opportunity for future research to explore their potential in reshaping digital health futures. These methods should not be a secondary focus, but specific BCD approaches or algorithms should be at the forefront of design when working with Black communities.

### Recommendations for Equity-Centered Digital Health Design With Black Communities

Findings from this review shed light on gaps and opportunities for consideration to better embed equity into the design of DHTs for Black communities. While well intentioned, designers, researchers, and engineers alike fall victim to superficial fixes and reproduce inequity; this has been a notable limitation of HCD and UCD [97]. To advance equity-oriented digital health innovation with Black communities, we must revisit the principles of BCD and transition from conceptual principles into actionable steps that practitioners can implement. Based on the findings of this review and existing BCD scholarship, we offer an operationalized, 5-step process recommendation to build on BCD tailored for digital health equity. These recommended steps provide a structured approach for centering Black lived experience, cultural context, structural determinants, and equity evaluation throughout the design lifecycle. Below we outline each step with a brief description, the BCD principles it aligns with, and guidance around what this recommendation can look like in practice drawn from included studies and current literature.

1. Engagement: During the early stages of the design process, designers should establish community partnerships and shared governance structures that position Black communities as collaborators in defining priorities, identifying health inequities factors, and codeveloping equity-focused success metrics. (Principles: 1/2/3).
  - Design practice: establishing community advisory boards [45]; partnering with trusted community organizations [45]; collaborating with community leaders within health care or cultural institutions [39].
2. Needs Assessment: Review the DDoH factors or other digital health equity metrics with an interdisciplinary team early (eg, community partners and social science/public health research), followed by an intersectional needs assessment focused on uncovering mismatch in cultural expectations and health care design [98]. Understand broader structural realities, including factors such as digital literacy, device access, stigma, immigration experiences, and culturally mediated health care expectations. (Principles: 2/3)
  - Design practice: identifying cultural-critical incidents through interviews, focus groups, or workshops; conducting a DDoH preanalysis early [31,93]; documenting barriers (eg, digital literacy gaps or device-sharing practices) [39].
3. Creation: Cocreate from a place of empowerment. Co-design activities should prioritize uncovered and newly identified design requirements by centering Black narratives, collective lived experiences, and speculative or future-oriented approaches that allow communities to imagine alternatives beyond existing systemic limitations. (Principles: 1/2/3)
  - Design practice: speculative design workshops [38,82]; Afrofuturist co-design sessions; narrative storytelling exercises to surface community visions for digital health technologies [38].
4. Development: Iterative prototyping should incorporate reflexive practices and explicit attention to power dynamics, ensuring that tensions, trade-offs, and cultural misalignments identified throughout the process are documented and revisited with community partners. (Principles: 1/2)
  - Design practice: maintaining reflexive design journals [99]; documenting design misalignments and revisiting them with community advisors; conducting iterative prototype reviews with community partners [52].
5. Evaluation: Finally, designers should evaluate through digital health equity accountability metrics. Evaluation should build upon traditional usability and acceptability metrics by revisiting the initial DDoH or digital health equity preanalysis. As a complementary evaluation approach, this will ensure that usability is accounted for, but that teams revisit equity within the process. (Principles: 2/3)
  - Design practice: revisiting shared goals between researchers and community partners; incorporating equity indicators into evaluation protocols [31]; documenting cultural-critical incidents during implementation to guide iterative improvements.

### Limitations

The goal of this study was to explore the current literature approaches design with Black communities. While the research team thoroughly engaged with the included articles, this review is not without limitations. The search strategy was developed using key terms identified as most relevant to the research questions; however, given the evolving and interdisciplinary terminology surrounding digital health design and equity-focused approaches, it is possible that relevant studies using alternative terminology were not captured, representing a limitation of this review. Furthermore, gray literature searches with tools such as Google Scholar can result in varying search results on the basis of the time of the search. This may have resulted in the research team possibly missing important studies that may have implications for designing with Black health equity in mind. Despite the explicit inclusion of terms related to equity design approaches, such as liberatory design, in our search criteria, this design approach did not appear in our search results. This may be due to the fact that this design approach is commonly used in the education sector, with limited applications in health technology at this time. Another consideration of note is that there may be systemic barriers at play that impact the research and use of equity-oriented design approaches, such as Afrofuturism within health care, as well as systemic factors impacting health equity research with Black communities in general. This may impact the quality and quantity of research that we see within this domain. Importantly, this review did not include a formal critical appraisal of included studies, consistent with the purpose of scoping reviews to map the breadth of existing evidence rather than assess study quality. Nevertheless, we observed variability in study size, rigor, and depth of reporting on the design and evaluation processes, which impacted the comparability and evaluation of each study and the interpretation of findings across studies. Finally, the concept of Blackness through a racialized lens is not equally applied across the globe and may be reductive or a more westernized approach to conceptualizing people of the African diaspora, which impacts the analysis of Black-centeredness and equity considerations. Taken together, this scoping review should serve as an initial step toward understanding the current landscape of BCD within the digital health domain.

### Conclusion

This review highlights the growing interest in designing DHTs for health equity concerns within the Black community but further underscores a critical need for clearer integration of equity within the design and evaluation process. Presently, studies that aim to address health disparities often vary widely in their application of design methods, consideration of equity within their design process, and few evaluate equity explicitly. Traditional design approaches, while common and pragmatic, tend to lack deeper equity analysis beyond the inclusion and engagement of Black communities. Explicitly BCD approaches such as Afrofuturism, although rich in potential, remain underused and vary in how they are operationalized in present-day digital health practices. A key takeaway from this review is the urgent need for well-defined, equity-focused design and evaluation criteria in digital health research, particularly when working with vulnerable communities. We call for an operationalization of BCD principles and an embedded consideration of the DDoH into the DHT design process. Through this work, we offer 5 guidelines aimed at assisting designers, researchers, and community health organizations with embedding and evaluating equity through the design cycle with Black communities. Future work must move beyond broad intentions and adopt design practices that are deliberate, inclusive, and justice-oriented when designing with Black communities and their intersecting cultural identities. Centering equity, not only in goals but in every step of the design approach, is essential to creating digital health tools that truly drive change.

## Supplementary material

10.2196/88995 Multimedia Appendix 1 Data sources and search strings.

10.2196/88995 Multimedia Appendix 2 Digital determinants of health analysis.

10.2196/88995 Checklist 1 PRISMA-ScR checklist.

## Abbreviations

BCD: Black-centered design
CBPR: community-based participatory research
DDoH: digital determinants of health
DHT: digital health technology
HCD: human-centered design
JBI: Joanna Briggs Institute
PRISMA: Preferred Reporting Items for Systematic Reviews and Meta-Analyses
PRISMA-S: Preferred Reporting Items for Systematic Reviews and Meta-Analyses Literature Search Extension Guidelines
PRISMA-ScR: Preferred Reporting Items for Systematic Reviews and Meta-Analyses Extension for Scoping Reviews
UCD: user-centered design
