# Supplementary material for: Equity-Oriented Design Processes and Evaluation of Digital Health Technologies for Black Communities Beyond Usability: Scoping Review
Source: J Med Internet Res. 2026 Jul 20;28:e88995. doi: 10.2196/88995 (PMC13384351; doi:10.2196/88995)
Supplement: Multimedia Appendix 1 [file jmir-v28-e88995-s001.docx]

| **Supplementary Material Search Strategy+** | | | |
| --- | --- | --- | --- |
| **Database Source** | **Search String** | **Advanced Search Feature** | **Date Last Searched** |
| **PubMed*** | ((( "health equity"[Mesh]) OR ("health inequities"[Mesh]) OR (health dispar* ) OR ( "healthcare disparities" ) OR ( "social determinants of health" ) OR ( "digital determinant of health" ) OR ( "structural racism" ) OR ( "systemic racism" ) OR ( "racial inequity" ) OR ( "health justice" ) OR ( "social justice" ) OR ("culturally appropriate") OR ("culturally sensitive") OR ("digital divide") OR ("digital health equity") OR ("low-resource settings") OR ("historically underserved")) AND (( " digital health"[Mesh]) OR ( digital health intervention* ) OR ( health technolog* ) OR ( "digital intervention" ) OR ( mHealth ) OR (m-health) OR ( "mobile health" ) OR ( eHealth ) OR (e-health) OR ("electronic health") OR ( telehealth ) OR ( telemedicine ) OR ( "virtual care" ) OR ( "remote care" ) OR ( telemonitor* ) OR ( remote monitor* ) OR ( "mobile app" ) OR ( "mobile application" ) OR ( "mobile health application" ) OR ( "health app" ) OR ( "health application" ) OR ( "web-based intervention" ) OR ( chatbot ) OR (chatbots) OR ( "conversational agent" ) OR ( "wearable technology" ) OR ( "artificial intelligence" ) OR (ai) OR ( "virtual reality" ) OR ("virtual agent") OR ( "augmented reality" ) OR ("medical information technology") OR ("health information technology") OR ("digital mental health") OR ("web-based platform"))) AND (( "human-centered" OR "human-centered design" ) OR ("user-centered" OR "user-centered design"[Mesh]) OR ( "person-centered design" ) OR ("patient-centered design") OR ( "participatory design" ) OR ( co-design ) OR ( codesign ) OR ("co-creation") OR ( "design thinking" ) OR ( "service design" ) OR ( "user-experience design" ) OR ( "UX design" ) OR ( "equity-centered design") OR ("equity centered community based design") OR ( "inclusive design" ) OR ( "design justice" ) OR ( "culturally responsive design" ) OR ( "Black-centered design" ) OR ( Afrofuturism ) OR ( "liberatory design" ) OR ( "speculative design" ) OR ( "critical design" ) OR ("science fiction prototyping") OR ("humanity centered design") OR ("Anti-Oppressive Design Framework") OR ("Anticipatory Ethnography") OR ( "universal design" ) OR ( "community based participatory design" OR "community based participatory research") OR ("community-based design") OR ("community engaged") OR ( "design for all" ) OR ("culturally tailored interventions" OR "culturally tailored") OR ( "equityxdesign" )) | None | April 2026 |
| **Scopus** | ( TITLE-ABS-KEY ( ( "health equity" ) OR ( health dispar* ) OR ( "healthcare disparities" ) OR ( "social determinants of health" ) OR ( "digital determinant of health" ) OR ( "structural racism" ) OR ( "systemic racism" ) OR ( "racial inequity" ) OR ( "health justice" ) OR ( "social justice" ) OR ( "culturally appropriate" ) OR ("culturally sensitive" ) OR ( "digital divide" ) OR ( "digital health equity" ) OR ( "low-resource settings" ) OR ("historically underserved" ) ) ) AND ( TITLE-ABS-KEY ( ( "human-centered" OR "human-centered design" ) OR ("user-centered" OR "user-centered design" ) OR ( "person-centered design" ) OR ( "patient-centered design" )OR ( "participatory design" ) OR ( co-design ) OR ( codesign ) OR ( "co-creation" ) OR ( "design thinking" ) OR ("service design" ) OR ( "user-experience design" ) OR ( "ux design" ) OR ( "equity-centered design" ) OR ( "equity centered community based design" ) OR ( "inclusive design" ) OR ( "design justice" ) OR ( "culturally responsive design" ) OR ( "black-centered design" ) OR ( afrofuturism ) OR ( "liberatory design" ) OR ( "speculative design") OR ( "critical design" ) OR ( "science fiction prototyping" ) OR ( "humanity centered design" ) OR ( "anti-oppressive design framework" ) OR ( "anticipatory ethnography" ) OR ( "universal design" ) OR ( "community based participatory design" ) OR ( "community-based design" ) OR ( "community engaged" ) OR ( "design for all" ) OR ( "culturally tailored intervention" OR "culturally tailored" ) OR ( "equityxdesign" ) ) ) AND ( TITLE-ABS-KEY ( ( " digital health" ) OR ( "digital health intervention" ) OR ( "health technology" ) OR ( "digital intervention" ) OR ( mhealth ) OR ( m-health ) OR ( "mobile health" ) OR ( ehealth ) OR ( e-health ) OR ("electronic health" ) OR ( telehealth ) OR ( telemedicine ) OR ( "virtual care" ) OR ( "remote care" ) OR (telemonitor* ) OR ( remote monitor* ) OR ( "mobile app" ) OR ( "mobile application" ) OR ( "mobile health application" ) OR ( "health app" ) OR ( "health application" ) OR ( "web-based intervention" ) OR ( chatbot ) OR( chatbots ) OR ( "conversational agent" ) OR ( "wearable technology" ) OR ( "artificial intelligence" ) OR ( ai )OR ( "virtual reality" ) OR ( "virtual agent" ) OR ( "augmented reality" ) OR ( "medical information technology") OR ( "health information technology" ) OR ( "digital mental health" ) OR ( "web-based platform" ) ) ) | Title-abs-key | April 2026 |
| **Web of Science** | ( "health equity" ) OR ( health dispar* ) OR ( "healthcare disparities" ) OR ( "social determinants of health" ) OR ( "digital determinant of health" ) OR ( "structural racism" ) OR ( "systemic racism" ) OR ( "racial inequity" ) OR ( "health justice" ) OR ( "social justice" ) OR ( "culturally appropriate" ) OR ("culturally sensitive" ) OR ( "digital divide" ) OR ( "digital health equity" ) OR ( "low-resource settings" ) OR ("historically underserved" ) (Topic) and ( " digital health" ) OR ( "digital health intervention" ) OR ( "health technology" ) OR ( "digital intervention" ) OR ( mhealth ) OR ( m-health ) OR ( "mobile health" ) OR ( ehealth ) OR ( e-health ) OR ("electronic health" ) OR ( telehealth ) OR ( telemedicine ) OR ( "virtual care" ) OR ( "remote care" ) OR (telemonitor* ) OR ( remote monitor* ) OR ( "mobile app" ) OR ( "mobile application" ) OR ( "mobile health application" ) OR ( "health app" ) OR ( "health application" ) OR ( "web-based intervention" ) OR ( chatbot ) OR( chatbots ) OR ( "conversational agent" ) OR ( "wearable technology" ) OR ( "artificial intelligence" ) OR ( ai )OR ( "virtual reality" ) OR ( "virtual agent" ) OR ( "augmented reality" ) OR ( "medical information technology") OR ( "health information technology" ) OR ( "digital mental health" ) OR ( "web-based platform" ) (Topic) and ( "human-centered" OR "human-centered design" ) OR ("user-centered" OR "user-centered design" ) OR ( "person-centered design" ) OR ( "patient-centered design" )OR ( "participatory design" ) OR ( co-design ) OR ( codesign ) OR ( "co-creation" ) OR ( "design thinking" ) OR ("service design" ) OR ( "user-experience design" ) OR ( "ux design" ) OR ( "equity-centered design" ) OR ( "equity centered community based design" ) OR ( "inclusive design" ) OR ( "design justice" ) OR ( "culturally responsive design" ) OR ( "black-centered design" ) OR ( afrofuturism ) OR ( "liberatory design" ) OR ( "speculative design") OR ( "critical design" ) OR ( "science fiction prototyping" ) OR ( "humanity centered design" ) OR ( "anti-oppressive design framework" ) OR ( "anticipatory ethnography" ) OR ( "universal design" ) OR ( "community based participatory design" ) OR ( "community-based design" ) OR ( "community engaged" ) OR ( "design for all" ) OR ( "culturally tailored intervention" OR "culturally tailored" ) OR ( "equityxdesign" ) (Topic) | Topic | April 2026 |

+No population-specific terms were included as the concept of Blackness may be described differently based on the geographic region. Instead, the search strings focused on digital health technology, health equity, and design approach key terms. *Where available, MeSH terms were utilized.
