# Supplementary material for: Equity-Oriented Design Processes and Evaluation of Digital Health Technologies for Black Communities Beyond Usability: Scoping Review
Source: J Med Internet Res. 2026 Jul 20;28:e88995. doi: 10.2196/88995 (PMC13384351; doi:10.2196/88995)
Supplement: Multimedia Appendix 2 [file jmir-v28-e88995-s002.docx]

| Supplementary Table: Digital Determinants of Health Analysis | | | | |
| --- | --- | --- | --- | --- |
| Author | **Individual** | **Interpersonal** | **Community** | **Societal** |
| Amore A. D. et al., 2023 [49] | Digital Self-efficacy, Attitudes Towards Use, Technology Access | Implicit Tech Bias, Patient-tech-clinician relationship | Community Partnerships, Community Tech Norms | Social Norms & Ideologies, Design Standards, Data Standards |
| Arueyingho et al 2025  [50] | Digital Self-efficacy, Attitudes Towards Use, Technology Access, Digital Literacy | Implicit Tech Bias, Patient-tech-clinician relationship | Community Partnerships, Community Tech Norms, Community Infrastructure | Data Standards, Social Norms & Ideologies, Design Standards, Algorithmic Bias |
| Baseman et al. 2025  [51] | Digital Self-efficacy, Attitudes Towards Use, Technology Access, Digital Literacy | Implicit Tech Bias, Patient-tech-clinician relationship | Community Partnerships, Community Tech Norms, Community Infrastructure | Social Norms & Ideologies, Data Standards |
| Ben-Zeev D et al. 2021  [52] | Attitudes Towards Use, Technology Access, Digital Literacy | N/A | Community Partnerships, Community Infrastructure, Community Tech Norms | Social Norms & Ideologies, Design Standards, Data Standards, Tech Policy |
| Blazey et al., 2023 [30] | Attitudes Towards Use, Digital Self-efficacy, Technology Access | Implicit Tech Bias | Community Infrastructure, Community Tech Norms | Social Norms & Ideologies |
| Boosley et al., 2022 [31] | Attitudes Towards Use, Digital Self-efficacy | Implicit Tech Bias | Community Tech Norms | Design Standards, Social Norms & Ideologies |
| Brewer et al.,  2023 [32] | Attitudes Towards Use, Digital Literacy, Digital Self-efficacy, Technology Access | Interdependence (Shared devices) | Community Infrastructure, Community Partnerships, Community Tech Norms | Design Standards, Social Norms & Ideologies |
| Bruns C 2021 [33] | Attitudes Towards Use, Technology Access, | Patient-tech-clinician relationship | Community Infrastructure, Community Partnerships, Community Tech Norms, Health Infrastructure | Design Standards, Tech Policy, Social Norms & Ideologies |
| Chandler R. et al., 2020 [34] | Attitudes Towards Use, Digital Literacy, Digital Self-efficacy, Technology Access | Implicit Tech Bias, Interdependence (Shared devices), Patient-tech-clinician relationship | Community Infrastructure, Community Partnerships, Community Tech Norms, Health Infrastructure | Data Standards, Design Standards, Social Norms & Ideologies, Tech Policy |
| Chandra et al. 2024 [59] | Attitudes Towards Use | N/A | Community Tech Norms, Community Partnerships | Social Norms & Ideologies |
| Clement et al. 2023  [60] | Technology Access, Attitudes Towards Use | Patient-tech-clinician relationship | Community Infrastructure, Community Tech Norms | Data Standards, Social Norms & Ideologies |
| Clifford et al.  [62] | Digital Literacy | Patient-tech-clinician relationship | Community Partnerships, Health Infrastructure | Data Standards, Social Norms & Ideologies, Tech Policy, Design Standards |
| Evans et al, 2016 [35] | Attitudes Towards Use, Digital Self-efficacy, Technology Access | Implicit Tech Bias, Patient-tech-clinician relationship | Community Partnerships, Community Tech Norms | Social Norms & Ideologies |
| Huang K.-T., 2024 [36] | Attitudes Towards Use, Digital Self-efficacy, Digital Literacy, Technology Access | Implicit Tech Bias | Community Infrastructure Community Partnerships, Community Tech Norms | Social Norms & Ideologies |
| Isler J. et al., 2019 [37] | Attitudes Towards Use, Digital Literacy, Digital Self-efficacy, Technology Access | Patient-tech-clinician relationship | Community Infrastructure, Community Tech Norms, Community Partnerships, Health Infrastructure, | Social Norms & Ideologies |
| Jefferson et al 2026  [63] | Attitudes Towards Use | Implicit Tech Bias | Community Partnerships | Social Norms & Ideologies |
| Le D. et al., 2018 [38] | Attitudes Towards Use, Digital Self-efficacy, Technology Access | N/A | Community Infrastructure, Community Partnerships, Community Tech Norms, Health Infrastructure, | Social Norms & Ideologies |
| Morse R. S. et al., 2021 [39] | Digital Literacy, Technology Access | Interdependence (Shared devices), Patient-tech-clinician relationship, | Community Infrastructure, Community Partnerships, Community Tech Norms, Health Infrastructure | Design Standards, Social Norms & Ideologies |
| Musumbulwa et al. 2026 [61] | Digital Literacy, Digital Self-efficacy, Technology Access, Attitudes Towards Use | Patient-tech-clinician relationship | Community Partnerships, Community Tech Norms, Health Infrastructure, Community Infrastructure | Social Norms & Ideologies, Data Standards, Design Standards |
| Newton Jr et al 2019 [58] | Technology Access | N/A | N/A | Social Norms & Ideologies, Design Standards |
| Nias, J. et al., 2022 [40] | Attitudes Towards Use, Digital Self-efficacy, Technology Access | Implicit Tech Bias | Community Infrastructure, Community Tech Norms | Algorithmic Bias, Data Standards, Design Standards, Social Norms & Ideologies, Tech Policy |
| Olajide et al 2026 [57] | Digital Literacy, Technology Access | Patient-tech-clinician relationship | N/A | Social Norms & Ideologies |
| Patchen L, et al., 2020 [41] | Attitudes Towards Use, Digital Literacy, Digital Self-efficacy, Technology Access | Implicit Tech Bias | Community Partnerships, Community Tech Norms | Social Norms & Ideologies |
| Plant A. et al., 2024 [42] | Attitudes Towards Use, Digital Self-efficacy, Technology Access | Implicit Tech Bias, Patient-tech-clinician relationship | Community Infrastructure, Community Partnerships, Community Tech Norms, Health Infrastructure | Data Standards, Design Standards, Social Norms & Ideologies, Tech Policy |
| Popowski et al 2025 [56] | Technology Access, Attitudes Towards Use | Patient-tech-clinician relationship | Health Infrastructure, Community Tech Norms | Social Norms & Ideologies, Data Standards |
| Thomson M. et al., 2024 [43] | Attitudes Towards Use, Digital Self-efficacy, Technology Access | N/A | Community Partnerships, Community Tech Norms | Data Standards, Design Standards, Social Norms & Ideologies, Tech Policy |
| Resnick D. et al., 2022 [44] | Attitudes Towards Use, Digital Self-efficacy, | N/A | N/A | Social Norms & Ideologies |
| Robles M. et al., 2022 [45] | Attitudes Towards Use, Digital Self-efficacy, Technology Access | Implicit Tech Bias, Interdependence (Shared devices), Patient-tech-clinician relationship | Community Tech Norms, Community Infrastructure | Data Standards, Design Standards, Social Norms & Ideologies, Tech Policy |
| Soehnchen et al 2023  [55] | Technology Access, Attitudes Towards Use | N/A | Community Tech Norms, Community Partnerships, Community Infrastructure, Health Infrastructure | Social Norms & Ideologies, Design Standards |
| Tesema N. et al., 2023 [46] | Attitudes Towards Use, Digital Literacy, Digital Self-efficacy, Technology Access | Implicit Tech Bias | Community Partnerships, Community Infrastructure | Design Standards, Social Norms & Ideologies |
| Veinot T. et al., 2013 [47] | Attitudes Towards Use, Digital Self-efficacy, Technology Access | Implicit Tech Bias, Interdependence (Shared devices), Patient-tech-clinician relationship | Community Partnerships, Community Tech Norms, Community Infrastructure | Data Standards, Design Standards, Social Norms & Ideologies, Tech Policy |
| Vilaro et al 2021  [54] | Attitudes Towards Use, Technology Access | Patient-tech-clinician relationship | Community Tech Norms | Social Norms & Ideologies, Design Standards |
| Williamson et al 2021  [53] | Technology Access, Attitudes Towards Use | N/A | Community Tech Norms, Community Partnerships, Community Infrastructure | Social Norms & Ideologies, Design Standards, Data Standards |
| Zhou E. et al., 2024 [48] | Attitudes Towards Use, Technology Access | Implicit Tech Bias | Community Infrastructure, Community Partnerships | Social Norms & Ideologies |
| N/A: not applicable | | | | |
